# Hypervirulent R20291 Clostridioides difficile spores show disinfection resilience to sodium hypochlorite despite structural changes

**DOI:** 10.1186/s12866-023-02787-z

**Published:** 2023-03-06

**Authors:** Dmitry Malyshev, Imogen Anne Jones, Matthew McKracken, Rasmus Öberg, Glenn M. Harper, Lovleen Tina Joshi, Magnus Andersson

**Affiliations:** 1 Department of Physics, Umeå University, Umeå, Sweden; 2 Faculty of Health, University of Plymouth, Plymouth, UK; 3 Umeå Centre for Microbial Research (UCMR), Umeå University, Umeå, Sweden

**Keywords:** Bacterial spores, Laser tweezers Raman spectroscopy, Raman spectroscopy, LTRS, C. difficile, Terbium

## Abstract

**Background:**

*Clostridioides difficile* is a spore forming bacterial species and the major causative agent of nosocomial gastrointestinal infections. *C. difficile* spores are highly resilient to disinfection methods and to prevent infection, common cleaning protocols use sodium hypochlorite solutions to decontaminate hospital surfaces and equipment. However, there is a balance between minimising the use of harmful chemicals to the environment and patients as well as the need to eliminate spores, which can have varying resistance properties between strains. In this work, we employ TEM imaging and Raman spectroscopy to analyse changes in spore physiology in response to sodium hypochlorite. We characterize different *C. difficile* clinical isolates and assess the chemical’s impact on spores’ biochemical composition. Changes in the biochemical composition can, in turn, change spores’ vibrational spectroscopic fingerprints, which can impact the possibility of detecting spores in a hospital using Raman based methods.

**Results:**

We found that the isolates show significantly different susceptibility to hypochlorite, with the R20291 strain, in particular, showing less than 1 log reduction in viability for a 0.5% hypochlorite treatment, far below typically reported values for *C. difficile*. While TEM and Raman spectra analysis of hypochlorite-treated spores revealed that some hypochlorite-exposed spores remained intact and not distinguishable from controls, most spores showed structural changes. These changes were prominent in *B. thuringiensis* spores than *C. difficile* spores.

**Conclusion:**

This study highlights the ability of certain *C. difficile* spores to survive practical disinfection exposure and the related changes in spore Raman spectra that can be seen after exposure. These findings are important to consider when designing practical disinfection protocols and vibrational-based detection methods to avoid a false-positive response when screening decontaminated areas.

**Graphical Abstract:**

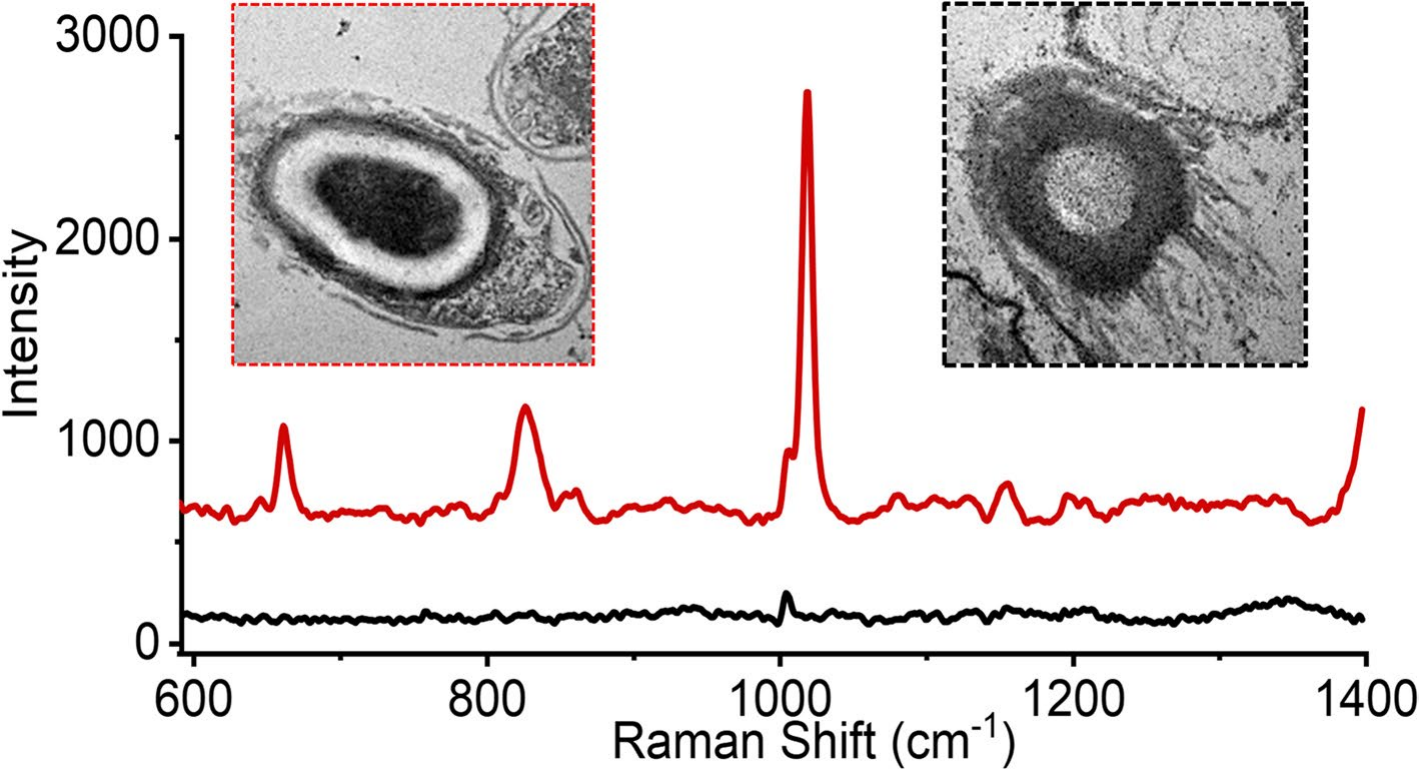

**Supplementary Information:**

The online version contains supplementary material available at 10.1186/s12866-023-02787-z.

## Background

*Clostridioides difficile* (also known as *Clostridium difficile*) is an anaerobic spore-forming bacterium and the most common cause of antibiotic-associated diarrhoea globally as well as the most common cause of healthcare-acquired infections (HCAI’s) in the USA [1]. *C. difficile* is normally harmless to healthy adults, for which ingestion of this common bacterium does not cause disease. Approximately 1-3% of the population are reported to be asymptomatic carriers, while in the remaining population, existing gut microbiota prevents colonization by *C. difficile* [2]. However, as a side effect of oral broad spectrum antibiotics, this microbiota can be depleted, allowing for colonisation with *C. difficile*. Hospitals are a hotspot for infections as they contain a high number of susceptible patients. Indeed, the health impact of *C. difficile* infection is huge. The economic costs for management of *C. difficile*-associated disease in US hospitals alone was estimated up to $6.3 billion per year [3]. In addition, *C. difficile* infection also has a 5.6-6.9% reported fatality rate leading to a significant loss of life [4].

The infectiveness of *C. difficile* and high outbreak management costs associated with *C. difficile* infections are due to the bacterium’s ability to form resilient endospores (spores). Spores can survive for months in the environment and cause infection when ingested. Spores are also capable of surviving many harsh conditions, such as $95^{\circ }$C wash cycles for hospital bedding and gowns [5]. Common hospital disinfection approaches such as alcohol-based hand wash, low-concentration sodium hypochlorite (bleach), and quaternary ammonium are ineffective at decontaminating spores on surfaces [6, 7]. Thus, hospitals need strict hygiene and cleanup protocols to prevent *C. difficile* outbreaks.

Therefore, to avoid *C. difficile* outbreaks, it is important to have rapid and specific detection techniques that can detect spores both in hospital facilities such as patient-specific rooms and also in laundry rooms. Since Raman spectroscopy is a non-invasive, label-free and highly specific technique that can provide a spectral fingerprint of a sample, both on surfaces or in a liquid, it has been proposed as one possible method. In addition, Raman spectroscopy has an advantage compared to other spectroscopic methods such as infrared (IR), since Raman signals are only moderately affected by the presence of water, so testing aqueous suspensions is easier. Also, Raman bands are significantly narrower and therefore easier to identify than fluorescence bands [8, 9]. As proof of concept, Raman spectroscopy has been used successfully to detect spores and also to track chemical changes in the spore body in time series. For example, Raman spectroscopy has been successfully used to identify and distinguish different pathogens [10], identify spore strains [11, 12], track the germination process [13, 14], and characterize the impact of disinfection chemicals on the spore body with time [15, 16].

Sodium hypochlorite is a common disinfection chemical used in hospitals and in homes [17]. It is therefore of interest to know if this chemical affects the Raman signature of *C. difficile* spores to avoid any false-positive response. The aim of this work is to compare the effect of sodium hypochlorite on three different *C. difficile* clinical isolates and characterize if the sodium hypochlorite concentrations impact their Raman spectra. Raman spectra were acquired of single spores using laser tweezers Raman spectroscopy (LTRS). These findings were then linked to structural changes in the spores assessed using transmission electron microscopy (TEM).

## Results and discussion

### *C. difficile* R20291 spores are highly resistant to disinfection with 0.5% sodium hypochlorite

Sodium hypochlorite is a common decontamination agent used widely around the world [7], being the main active ingredient in household and industrial bleach. Sodium hypochlorite is a chlorine releasing agent (CRA) and works by degrading organic material in several reactions: saponification of fatty acids and neutralization and chloramination of amino acids [7, 18]. This will degrade the spore structure if exposed for long time scales or at high concentrations [16]. Spores of *C. difficile* were previously reported to be quite resilient to decontamination with lower concentrations of hypochlorite [19]. This is particularly important since, in a practical cleaning environment, spores might only be exposed for a shorter duration due to issues with cleaning protocols and time pressure for healthcare workers [20] or the concentration of sodium hypochlorite can be too low for effective decontamination [21]. Age and storage conditions of the hypochlorite can also affect the remaining active chlorine and, thus, its efficiency at decontaminating surfaces. Hospitals often use a 1:10 dilution of bleach (approximately 0.5% or 5000 ppm) but reported spore disinfection varies [22, 23]. Decontamination of *C. difficile* with hypochlorite was previously reported at 4.3 log (0.5%, 10 min) [19], 5.7 log (0.6%, 10 min) [24], 6 log (0.5%, <10 min) [25] and 4.64 - 5.39 log (0.5%, 5 min) [26]. This is a substantial variance (4.3 - 6 log) across studies despite similar conditions. In addition, it has been noted that decontamination is also pH-dependent and at physiological pH, the efficiency of hypochlorite is reduced to <1 log (0.5%, 10 min) [27]. This is in contrast to *Bacillus* species, where sodium hypochlorite was reported to be more effective at decontaminating spores at physiological pH [28], and even completely degrading spores at concentrations of 0.2% [29].

**Fig. 1:**
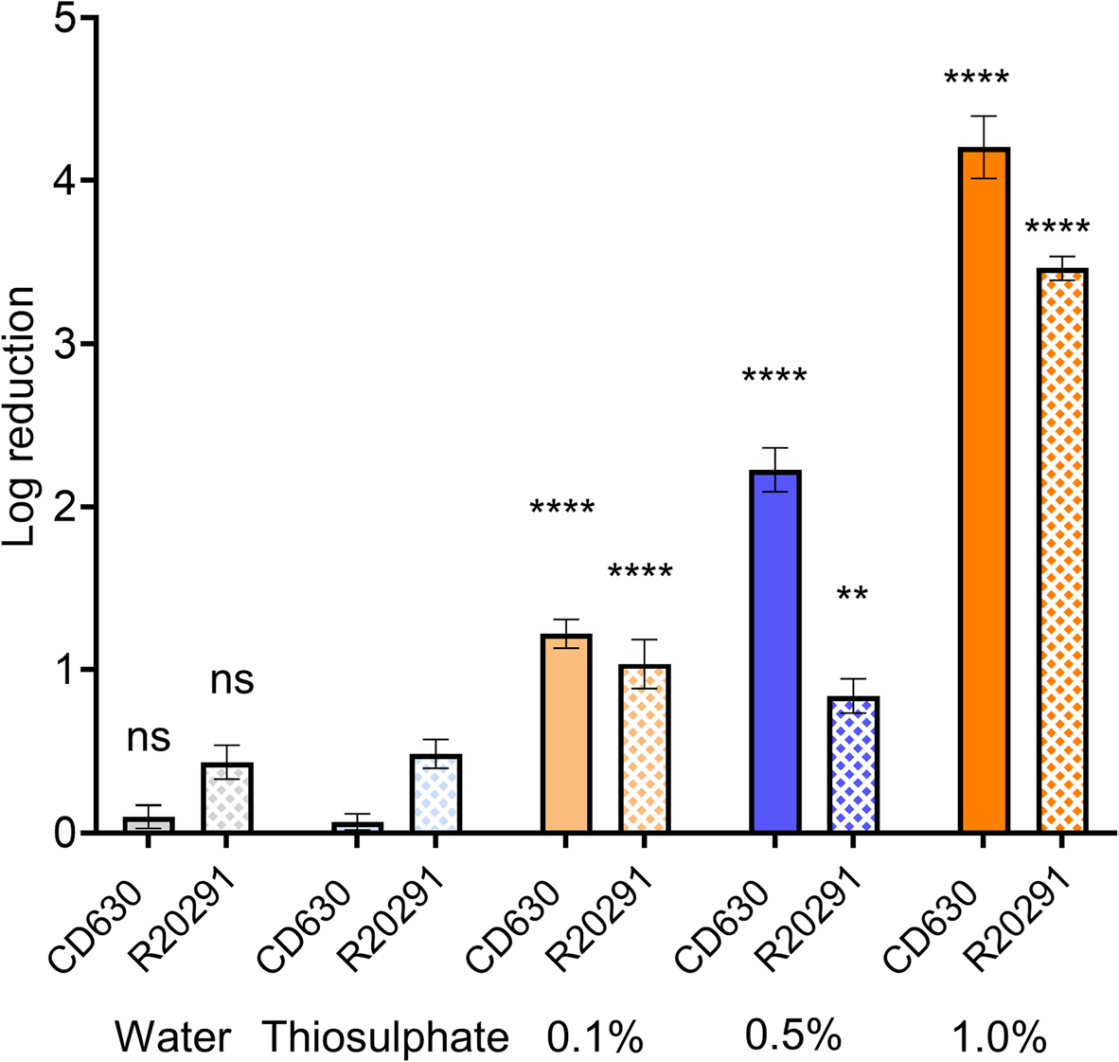
Viability of *C. difficile* CD630 and R20291 spores following hypochlorite treatment. Spores were incubated with 0.1%, 0.5% and 1.0% hypochlorite for 10 min. Controls with water and thiosulphate alone are also shown. The R20291 strain, in particular, is very resilient to low concentrations below 1.0% hypochlorite. Stars indicate the statistical significance of the reduction compared to the respective water control, with 2 stars for p $\le$ 0.01 and 4 stars for p $\le$ 0.0001; “ns” indicates no significance

We found DS1813 *C. difficile* spores to be in line with previously reported results for spores with 5.1 ± 0.2 log reduction after 0.5% hypochlorite treatment for 10 min, see Fig. S1. By contrast, the CD630 and the R20291 were more resilient than previously reported in the literature, with a decontamination in 0.5% hypochlorite causing a 2.2 ± 0.1 log reduction in the CD630 strain, and 0.8 ± 0.1 reduction in the R20291 strain compared to control. This is significantly below the requirements for surface decontamination for an area in clinical environments, where usually at least 4 log is expected [30]. We, therefore, made a viability assay in which the concentration was varied between 0.1%, 0.5%, and 1.0%, with the results shown in Fig. 1.

One clear observation is that the resistance to hypochlorite is much higher than previously reported for the R20291 specifically. Survival of viable spores and resistance to standard concentrations of biocide was previously reported in *C. difficile* treated with sodium dichloroisocyanurate, a different CRA [21]. A somewhat higher resistance to hypochlorite for R20291 compared to NCTC 12727 was recorded by Siani *at al.,* [26], but not the level we observed. The emergence of bacterial biocide tolerance has become a concern in recent years with the overuse of disinfectants due to the COVID-19 pandemic [31]. As CRAs have been used extensively over the past two decades to treat surfaces against *C. difficile* [20, 32], it is possible it has resulted in the evolution of biocide tolerance in *C. difficile*, specifically in hypervirulent R20291. However, this warrants further investigation to determine whether this matches trends in biocide tolerance of *C. difficile* persisting within clinical environments. This hypothesis aligns with the final conclusions in Dawson et al., 2011 that the type of disinfectant used should be carefully considered before deployment for decontamination of surfaces [23].

**Fig. 2:**
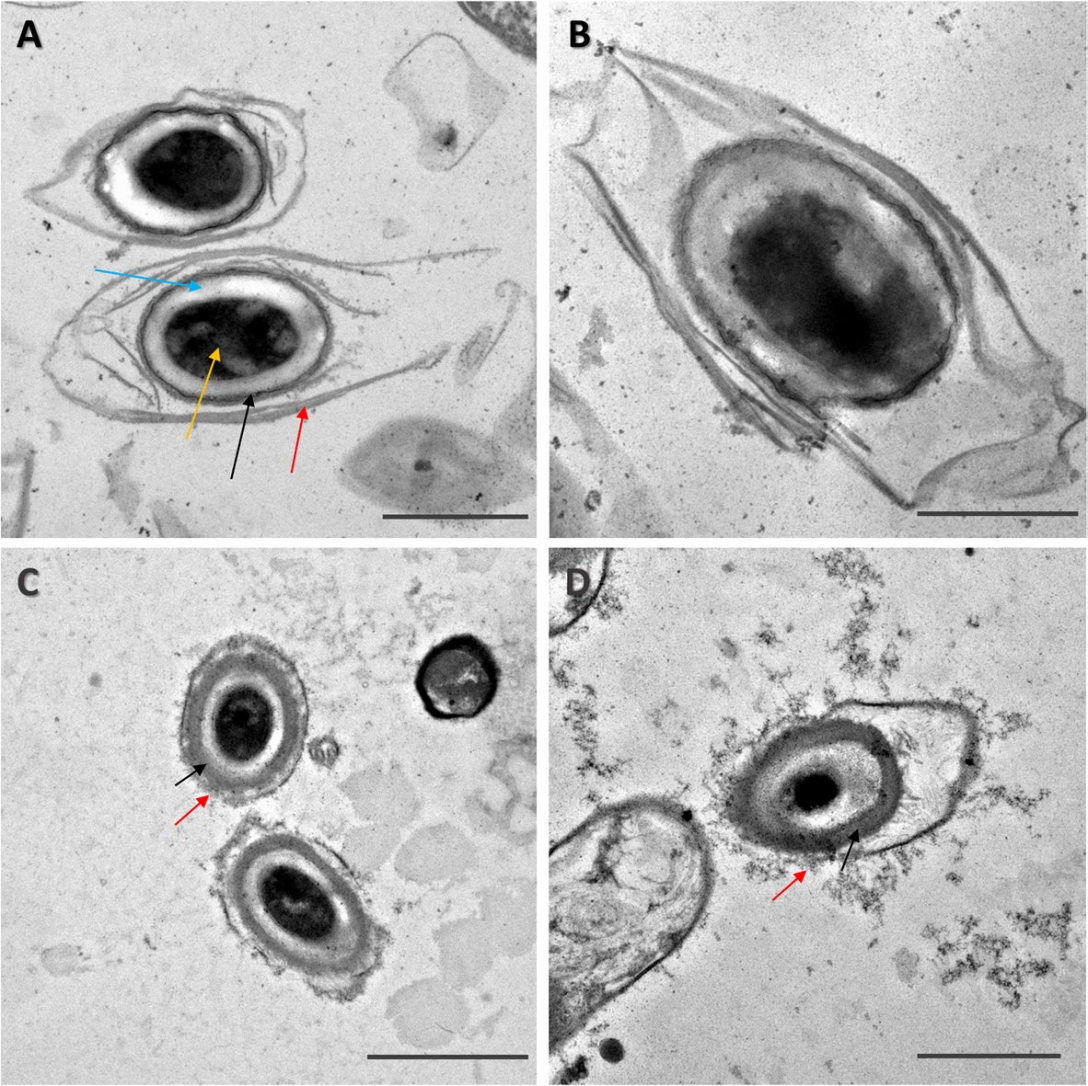
Representative TEM images showing the effect of sodium hypochlorite treatment on *C. difficile* spores. Untreated spores are shown on **A** (R20291) and **B** (CD630). These spores are intact, with the exosporium (red), coat (black), cortex (blue) and core (gold arrow) layers clearly defined and intact. The spores appear disrupted after a 1% sodium hypochlorite treatment, with the exosporium and coat layers no longer intact in both **C** (R20291) and **D** (CD630). Some spores no longer have an electron-dense core. Scale bars are 1 $\upmu$m

**Fig. 3:**
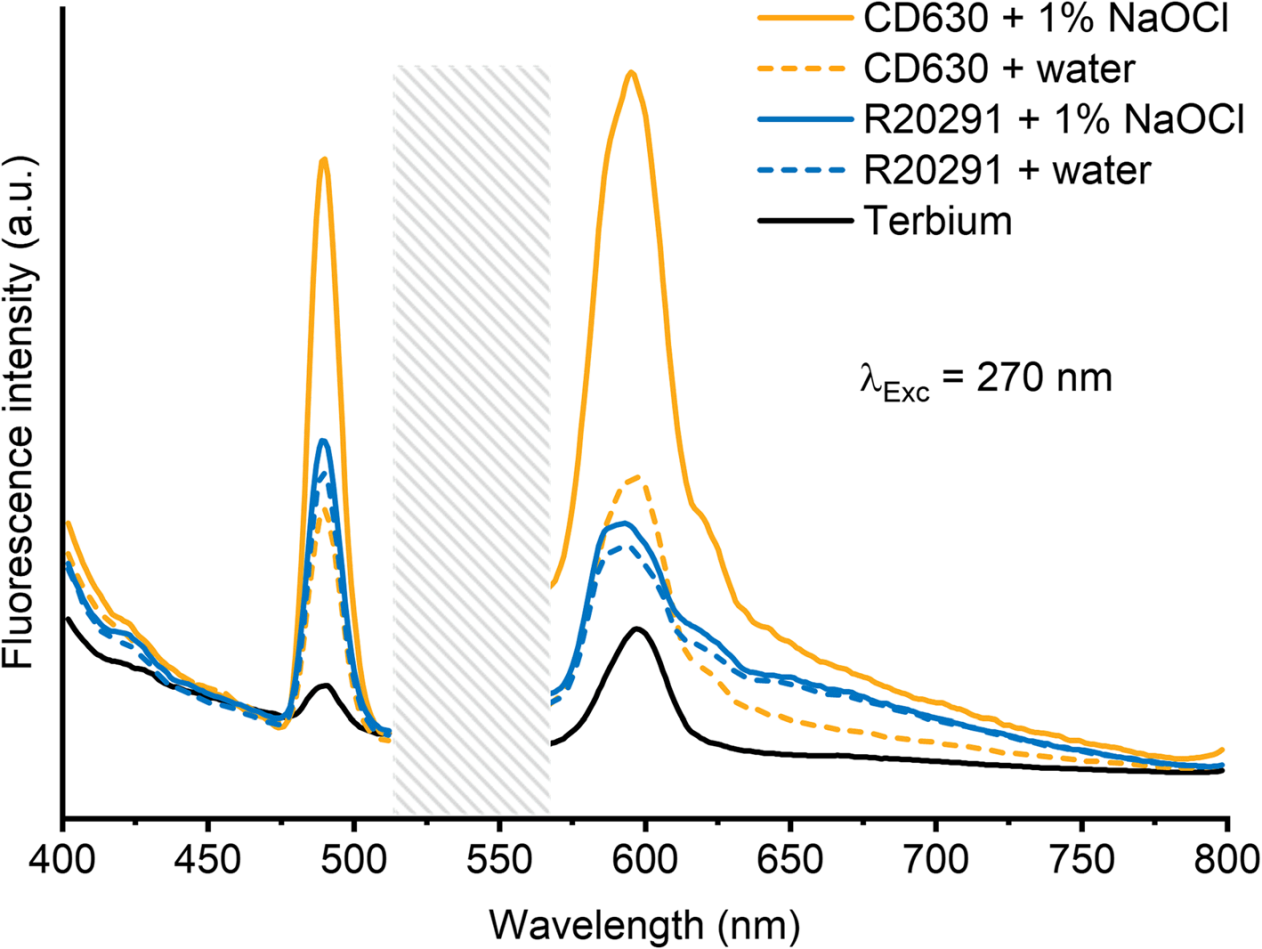
Fluorescence emission spectra from supernatants after centrifugation of CD630 (orange) and R20291 (blue), both controls (dashed) and exposed to sodium hypochlorite (solid lines). After excitation at 270 nm, we observed fluorescence emission at 490 nm and 595 nm. Due to excessive interference from second order scattering saturating the detector in the span 520 - 560 nm, this area has been shaded for clarity

To assess the disruption of spores after exposure to sodium hypochlorite we used TEM imaging. A micrograph of spores at the higher 1%, 10 min hypochlorite exposure is shown in Fig. 2, and additional fields of view are shown in Fig. S2-S3. Unexposed control spores (Fig. 2A-B) are intact with the core (gold), cortex (blue), coat (black) and exosporium (red arrows) clearly visible. Spores treated with 1% hypochlorite have a degraded or missing exosporium and a less electron-dense coat (Fig. 2C-D). We further checked whether the spore degradation coincides with release of spore core materials by measuring the content of calcium dipicolinic acid (CaDPA) released by the spore. We measured the DPA content of the supernatant of spore suspensions using fluorescence spectroscopy. Using the same concentration of spores and water as a buffer ensures that the fluorescence response of DPA is not affected by changes in pH [33]. The DPA signal from the spores was enhanced using terbium, which makes a complex with DPA [34, 35], boosting its fluorescence yield. As shown in Fig. 3, when exciting the solution at 270 nm we observe fluorescence emission at 490 nm and 595 nm that is higher than the reference terbium signal for CD630 and R20291 supernatants, both in controls and in 1% sodium hypochlorite-treated spores. For the CD630 sample exposed to hypochlorite, however, we observe a fluorescence signal that is more than double in relative intensity compared to the other supernatants, suggesting a higher DPA-concentration. This further suggests a higher level of susceptibility to sodium hypochlorite for CD630 compared to the hypervirulent R20291 strain.

There are also some spores that appear as pale outlines with completely unstained internal content, similar to what we have previously observed in *Bacillus thuringiensis* spores [16] treated with 0.5% sodium hypochlorite for 30 min. This visual disruption was linked with the loss of the spore’s store of CaDPA and in the more disrupted cases, the loss of most internal material. To quantify the changes in the spores with high accuracy, we turned to single spore analysis using LTRS.

### Raman spectra of decontaminated spores fall into distinct groups

**Fig. 4:**
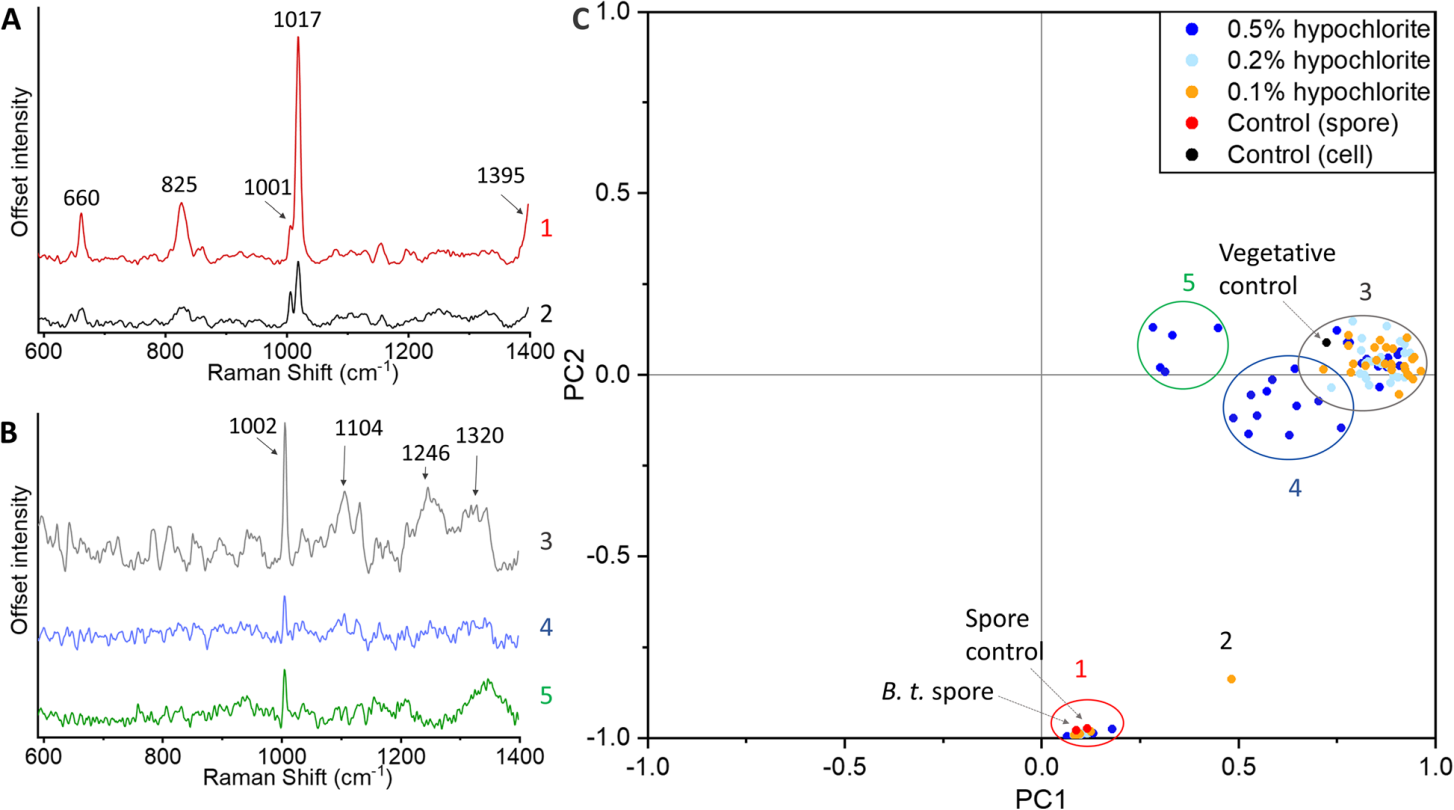
Representative observed spectra of spores with CaDPA (**A**) and without CaDPA (**B**) in the 600 - 1400 cm$^{-1}$ spectral range. Principal component analysis (PCA) of sodium hypochlorite-treated spores (n=90). The numbering of the spectra corresponds to the numbering of the groups in the PCA plot (**C**). Untreated control spore, live vegetative cell and a *B. thuringiensis* spore are also shown on the PCA plot for reference (marked with arrows)

Using the LTRS instrument we optically trapped individual spores and analysed their Raman spectra to quantify the chemical content. This approach provides a spectral fingerprint of the trapped object and can thereby measure chemical differences between hypochlorite-treated and untreated spores, as well as differences with vegetative cells. A typical Raman spectrum of a *C. difficile* spore in the 600-1400 cm$^{-1}$ spectral range is shown in Fig. 4A, with major peaks at 660, 825, 1001, 1017 and 1395 cm$^{-1}$. This is in line with the Raman spectrum of *C. difficile* spores described in literature [10, 36, 37]. The spectrum is dominated by the Raman peaks of CaDPA, a key chemical in the spore’s wet heat resistance that makes up to 25% of the dry weight of the spore core. The reported peaks of CaDPA are 660, 825, 1017, 1395 and 1575 cm$^{-1}$, while the 1001 cm$^{-1}$ is due to phenylalanine [10, 36, 38, 39]. During germination, or if the spore body is damaged, the intensity of the CaDPA Raman peaks are significantly reduced or disappear completely as the spore loses its CaDPA store. Once started, the process takes only $\sim$30 s with hypochlorite-induced CaDPA loss, faster than normal germination [16]. The Raman spectrum of spores in the 1400-1700 cm$^{-1}$ range, which is outside the range of Fig. 4, is shown in Fig. S4A. In total, we acquired n = 6 spore spectra of untreated spores, all show consistent spectra.

While some hypochlorite-treated spores appeared similar to the control spores, some spores produced different spectra, see Figs. 4B and S4B. These spores are missing CaDPA related peaks. This loss of CaDPA is expected as it was previously reported that hypochlorite-treated spores will release CaDPA [28, 40]. This is also consistent with TEM images in Fig. 2, showing some spores missing their electron-dense core, and fluorescence measurements in Fig. 3, indicating that DPA leaked out from the spore. In addition to this lack of CaDPA, we see that there is variation in the other peaks among spectra in these spores, and with different prominence of different peaks. The main conserved peak among all spores is the 1001 cm$^{-1}$ peak associated with phenylalanine, a structural amino acid in both spores and vegetative cells. A peak at 1101 cm$^{-1}$ and a double peak at 1246-1320 cm$^{-1}$ are also present.

To compare spectra of different groups of spores/cells we quantified the differences between all of the measured spores with principal component analysis (PCA). PCA is a suitable method to compare complex data such as spectra to each other and quantify differences between them [41]. PCA has previously been used to compare Raman spectra of explosive chemicals [42], and to compare blood plasma spectra when searching for viral biomarkers [43]. As such, this method was suitable for comparing the differences in the Raman spectra in the spores. The results from this analysis is shown in Fig. 4C for the 600 - 1400 cm$^{-1}$ range spectra (n=90, 10 for each experimental condition), with representative spectra from each group in Fig. 4A-B corresponding to the circled groups. The results for the 1000 - 1700 cm$^{-1}$ range (n=90) are included in Fig. S4C. Spores that retain the CaDPA peak make a compact cluster (Group 1) with 10 of 90 spores (11%) in this cluster. Untreated control spores, both *C. difficile* and *B. thuringiensis*, also fall into Group 1, in line with earlier research that disinfection of bacterial spores does not necessarily lead to changes in the Raman spectrum [44]. A single outlier, marked as Group 2, also has CaDPA peaks. This outlier is a spore with reduced CaDPA peak prominence, which is otherwise spectrally similar to other CaDPA-containing spores.

The remaining spores lack CaDPA peaks and fall into three groups. Many spores retain their Raman peaks, except CaDPA. This is the most common group of spores among those observed (63 of 90 spores, 70%) and has been labeled as Group 3. Spores in this cluster retain their Raman spectra, such as the peaks at 1104 and 1246 cm$^{-1}$, and their similarity to Group 1 spectra can be seen in Fig. S5, where the two spectra are shown on the same scale. *Bacillus subtilis* spores have been previously shown to release their CaDPA after hypochlorite treatment [16], resulting in similar spectra to Group 3. Spectra in this group are also similar to those from vegetative cells, as shown by the marked vegetative control spectrum. These spectra are also similar to other vegetative cell spectra, with Raman peaks at 1101, 1245 and 1319 cm$^{-1}$ [10].

Group 4 spores (13 of 90 spores, 14%) partially overlap with Group 3 (2 overlapping spores), and consist of spores with reduced peaks, including the phenylalanine peak. These spores are likely the broken-down degraded spores that can be observed under TEM as pale outlines, having lost both the core content and much protein content, with the remaining content accounting for the smaller phenylalanine peak. Group 5 is the smallest group (5 of 90 spores, 5%), and is an outlier to all the other groups. These spores have a small 1001 cm$^{-1}$ phenylalanine peak, and a lack of other prominent peaks, similar to Group 4 spores. However, the spores in Group 5 also contain an additional broad peak centered at 1350 cm$^{-1}$. It is not a peak that is seen in spores, vegetative cells or in sodium hypochlorite. It is outside the Amide III band, which is usually placed at 1200-1300 cm$^{-1}$ [45]. It is also not a peak seen in the subtracted background. A possible assignment of this peak is tryptophan and thymine from aggregates on the surface of the spore from lysed spore fragments [46].

We did not see any clinical isolate dependent distribution among the spectra (Fig. S6), with all clinical isolates being present in all groups in the principal component analysis. Despite differing levels of resistance to sodium hypochlorite as well as differences in virulence and structure [47, 48], there were no changes visible under Raman spectroscopy between the strains. While Stockel et al., have shown that it is possible to tell apart different *Bacillus* spore species with Raman spectroscopy [11, 12], it was with intact, and not chemically degraded spores. In addition, the method requires creating an extensive reference library with $\sim$ 1,000 spores of each strain to be able reliably differentiate the spores.

We did, however, see spore spectra grouping based on the hypochlorite concentration used. All 5 spores in Group 5 and 10 of the 13 spores in Group 4 were spores treated with 0.5% hypochlorite. This is consistent with the prediction that higher concentrations of sodium hypochlorite will lead to greater spore degradation, and more lysed components in solution. However, there were other 0.5% hypochlorite-treated spores in Groups 1 and 3, with the same spectra as untreated controls. The observation that some spores remain visually and structurally intact despite extremely aggressive conditions like 0.5% hypochlorite highlights the difficulty of decontaminating spores. This is compounded by the fact that spores can clump together [49], shielding the innermost parts of the clump from the chemicals. Finally, spores display variance even within the same population, including in coat and exosporium thickness [50], potentially allowing spores with thicker layers to be more resilient to decontamination.

### *C. difficile* show higher resilience to hypochlorite than *B. thuringiensis*

The variations in the resistance properties of *B. thuringiensis* and *C. difficile* may lie in their structural differences or response to sodium hypochlorite. As noted in Fig. 2, *C. difficile* spores show degradation of their outer layers, but many retain their electron dense core (with electron density correlated to the internal CaDPA) [50–52].

**Fig. 5:**
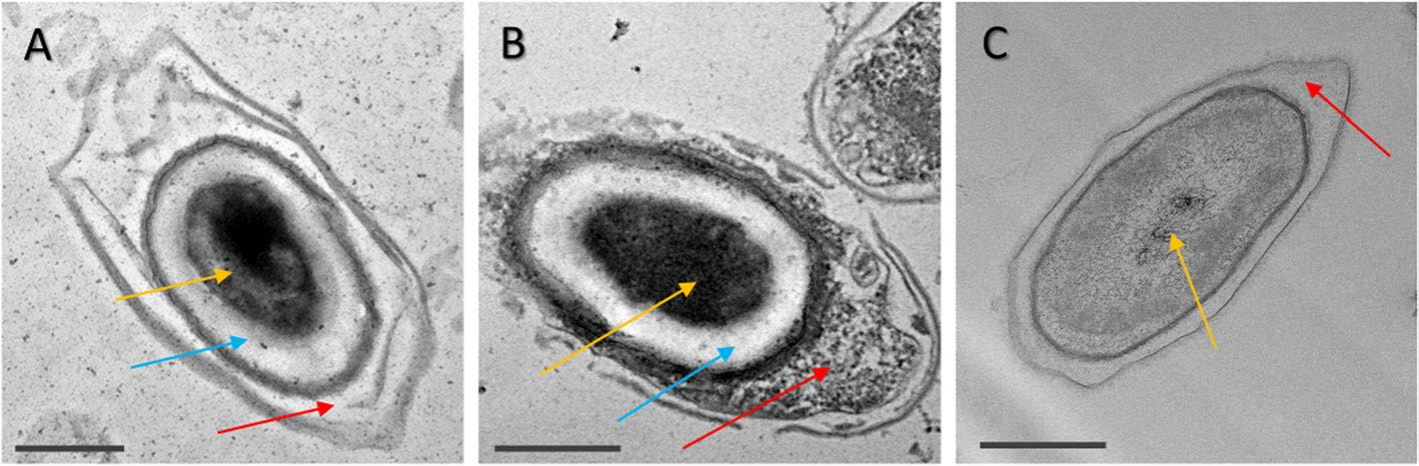
Representative TEM micro graphs of *C. difficile* R20291 (**A**) and CD630 (**B**) spores, and *B. thuringiensis* (**C**) spores treated with 0.5% sodium hypochlorite for 10 minutes. In R20291, many spores remain visually intact, with the core (yellow arrows) remaining dense, the cortex (blue arrow), being clear and visible. In CD630, while the core and cortex are still intact, there is leakage of material from the coat into the interspace between the coat and exosporium (red arrow). In *B. thuringiensis*, the core loses electron density and cortex is no longer visible after treatment. Scale bars are 500 nm

We compared this structural appearance to that of *B. thuringiensis*. We chose identical conditions of a 10 min treatment with 0.5% sodium hypochlorite for each species. As shown in Fig. 5, the appearance of spores differs markedly. Most spores of *C. difficile* R20291 appeared visually intact, with the core, cortex, coat and exosporium appearing whole. Spores of *C. difficile* CD630 strain also had an intact core and cortex, but electron dense material appeared in the interspace between the coat and the exosporium, while the coat exhibited pronounced structural changes. By contrast, most spores of *B. thuringiensis* had a different appearance, with the core and cortex melding into no longer having a visible boundary (additional representative fields of view are shown in Fig. S7-S10). This difference is of interest as it indicates that there are potential mechanistic differences in the way the spores respond to hypochlorite. Although in both species, spores release their core content, as previously shown in Fig. 4, *B. thuringiensis* spores appear to go through a process of core content leaching into the cortex. It was previously reported that spore CaDPA release is a rapid process (of the order of less than minute) [16, 40] and does not happen simultaneously, so the appearance of the *B. thuringiensis* spores indicate there is a stage before CaDPA is released, as it permeates into the cortex.

## Conclusions

Sodium hypochlorite is a common cleaning agent, both in homes and hospitals. In this work, we looked at changes in spore physiology of *C. difficile* clinical isolates in response to sodium hypochlorite treatment. We found that sodium hypochlorite inactivates *C. difficile* spores with rates of 0.8 log to 5.2 log (with 0.5%, 10 minute exposure) depending on clinical isolate, with the R20291 being the most resilient to disinfection. Interestingly, even at concentrations typically used in clinical environments, we found that hypochlorite is largely ineffective against R20291. TEM image analysis suggests that R20291 spores have higher structural resilience to sodium hypochlorite than the other analysed strains. This is supported by fluorescence spectroscopy, showing lower amounts of DPA leakage in the hypervirulent R20291 strain compared to CD630.

To quantify spores’ biochemical change in response to treatment and to find out if Raman spectroscopy is a robust method to distinguish between untreated and disinfected spores, we employed Laser Tweezer Raman spectroscopy on single spores. We compared the chemical content and composition of spores and found that spore spectra fall into distinct groups. These groups are related to the amount of spore degradation and similarity to vegetative cells. However, there is no specificity to spore strain. Despite strain, the majority of the spores (70%) lost their CaDPA-related Raman peaks upon disinfection. Disinfected spores that retain their CaDPA-related Raman peaks are not distinguishable from controls. Thus, relying on CaDPA as a biomarker, or other spore biochemicals, for vibrational detection and viability assessment can be risky since there is a high probability of a false-positive response.

## Methods

### Strains, culture media, and conditions

*C. difficile* isolate DS1813, CD630 and R20291 spores were sourced from the Anaerobic Reference Unit, University Hospital Wales, Cardiff, UK [51]. All three isolates are clinical isolates, with the DS1813 and R20291 belonging to the hypervirulent 027 ribotype of *C. difficile*, while the CD630 belong to the 012 ribotype and is a commonly studied and fully gene sequenced [53]. Spores were grown on BHIS-ST agar (BHI supplemented with 0.5% yeast extract, 1% L-cysteine and 0.1% sodium taurocholate) [54] at 37$^{\circ }$C for 4 days under anaerobic conditions (85% N$_{2}$, 10% CO$_{2}$, 5% H$_{2}$). The colonies were collected, washed with deionised water and left overnight at 4$^{\circ }$C to release of spores from mother cells. The suspensions were then purified using non-damaging density gradient centrifugation in 50% sucrose as described previously [21, 55, 56]. Spores were then washed in deionised water and stored at 4$^{\circ }$C. This method avoids spore purification steps such as lysozyme or proteinase, to ensure that spores and their resilience to chemicals are representative of the spores typically found in hospital environments [57]. *B. thuringiensis* (ATCC 35646) spores were sourced from the Swedish Defence Research Agency (FOI), Umeå, Sweden.

We determined the concentration of viable spores in the stock by serially diluting in deionised water down to $10^{-7}$ concentration and 10 $\upmu$l drops plated [58] onto BHIS-ST agar plates and grown at 37$^{\circ }$C in anaerobic conditions.

### Spore biocide treatment

We sourced the sodium hypochlorite solution used in the experiments from Merck (105614, Sigma Aldrich), stored at 4$^{\circ }$C and diluted as appropriate for the experiments.

Each of the 3 isolates was decontaminated with 0.1%, 0.5% and 1.0% sodium hypochlorite (1,000, 5,000 and 10,000 ppm active chlorine), for 9 experimental sets in total. The decontamination procedure for each sample was as follows. 100 $\upmu$l of spore suspension in water at a concentration of $10^{9}$ spores/ml was mixed with 100 $\upmu$l of double concentrated NaOCl (0.2%, 1.0% and 2.0%) and left for 10 minutes. The biocide was then neutralised with 0.5% sodium thiosulphate as described previously [21]. The spores were then washed with deionised water, by centrifuging and discarding the supernatant twice, to remove reacted chemicals.

The reduction in viable spore count was determined by spreading 100 $\upmu$l culture onto BHIS-ST plates. Spores were grown in anaerobic conditions at 37$^{\circ }$C for 48 hours and colonies were counted from a plate with appropriate dilution. There were 3 biological replicates for each experimental set.

### Fluorescence spectroscopy

Spore suspensions with a starting volume of 1 ml and an initial concentration of $10^{8}$ spores/ml were treated with hypochlorite as described above. The neutralised suspensions and the controls were centrifuged to pellet the spores and organic debris, and the supernatant was collected. We then mixed terbium chloride (439657, Sigma Aldrich) into the supernatant solutions at a 1:1 molar ratio to the total amount of DPA in the spores as described by Kocisova et al. [59], being $\sim$ 10$^{-4}$ M terbium in a 3 ml volume for $10^{8}$ spores. We diluted this mixture by a factor of 10 in deionised water to achieve appropriate optical density $\sim$ 0.1 around the fluorescence excitation wavelength 270 nm. We then measured the fluorescence of 3 ml solutions (n=3 technical replicates) placed in quartz glass cuvettes (6610001200, Agilent Technologies) using a fluorescence spectrophotometer (Cary Eclipse, Agilent Technologies). The solutions were excited at 270 nm with emission measured at 400 - 800 nm. Data was then converted into a numerical format and processed in Origin.

### Sample preparation and reference spectrum acquisition

We prepared a sample by placing a 1 cm diameter ring of 1 mm thick vacuum grease on a 24 mm $\times$ 60 mm glass coverslip. We added 5 $\upmu$l of the spore suspension into the ring, after which we sealed it by placing a 23 mm $\times$ 23 mm glass coverslip on top. After the sample was placed in the LTRS instrument, we measured the Raman spectra of the spores using 2 accumulations of 10 seconds. We measured 20 individual spores for each sample (10 measurements in the 600-1400 cm$^{-1}$ and 10 in the 1000-1700 cm$^{-1}$ range), for 180 measurements in total. There were also triplicate controls at each spectral range. The background spectrum of the spore suspension was also measured and subtracted.

### Experimental setup and measurement procedure

We acquired Raman spectra from spores using our custom-built LTRS instrument. The instrument is built around an inverted microscope (IX71, Olympus) [44, 60]. We used a Gaussian laser beam operating at 785 nm (Cobolt 08-NLD) that is coupled into the microscope using a dichroic shortpass mirror with a cut-off wavelength of 650 nm (DMSP650, Thorlabs). Imaging and focusing of the beam were achieved by a 60$\times$ water immersion objective (UPlanSApo60xWIR, Olympus) with a numerical aperture of 1.2 and a working distance of 0.28 mm. The same laser was used for Raman light excitation. In general, we operated the laser at a fixed output power of 100 mW corresponding to a power of about 60 mW in the sample (total energy of 1.2 J when exposed for 20 seconds). This power chosen was well below those previously recorded to damage spores [61, 62].

We collected the backscattered light by the microscope objective and passed it through a notch filter (NF785-33, Thorlabs) to reduce the Rayleigh scattered laser line. Further, to increase the signal-to-noise ratio, we mounted a 150 $\upmu$m diameter pinhole in the focal point of the telescope. The filtered light was coupled into our spectrometer (Model 207, McPherson) through a 150 $\upmu$m wide entrance slit where a 600 grooves/mm holographic grating disperses the light [63]. The Raman spectrum was then captured using a Peltier cooled CCD detector (Newton 920N-BR-DDXW-RECR, Andor) operated at -95$^{\circ }$C. Our system has a Raman wavenumber spectral resolution of < 3 cm$^{-1}$ and accuracy of $\sim$ 3 cm$^{-1}$.

### Data processing and analysis for reference spectra

The statistical significances of spore decontamination results were calculated using two-way Anova with Dunnett’s multiple comparisons test, done in Graphpad Prism 9 (Prism 9.3, GraphPad Software). Data normality was confirmed with a QQ plot and with the Kolmogorov Smirnov test. We used an open-source Matlab script (Matlab R2022, Mathworks) provided by the Vibrational Spectroscopy Core Facility at Umeå University to process Raman spectra [64]. To baseline correct the spectra we used an asymmetrical least-squares algorithm [65] with $\lambda$ = 10$^5$ and p = 10$^{-3}$. We smoothed spectra using a Savitzky-Golay filter [66] of polynomial order 1 and a frame rate of 5. Principal component analysis of the spectra (PCA) was carried out in Graphpad Prism 9. Data was mean-centered and PCA was based on the correlation matrix. Graphs were plotted in Origin 2018 (OriginLab).

### Electron microscopy

Samples for TEM were prepared as liquid suspensions of spores after treatment with sodium hypochlorite and neutralisation with thiosulphate, while untreated control samples were suspended in water. After the incubation, samples are centrifuged and resuspended in MQ water twice to wash off any aqueous chemicals. Spores are fixed with 2.5% glutaraldehyde (TAAB Laboratories, Aldermaston, England) in 0.1 M sodium cacodylate buffer and further postfixed in 1% aqueous osmium tetroxide. They are further dehydrated through an ethanol concentration series and finally embedded in Spurr’s resin (TAAB Laboratories, Aldermaston, England) and polymerised overnight at 60 °C. 70 nm ultrathin sections are then post contrasted in uranyl acetate and Reynolds lead citrate. *C. difficile* spores were imaged using a JEM 1400 (JEOL Ltd.) using a Orius camera (Gatan Inc.). *B. thuringiensis* spores were imaged using a Talos L120C (FEI, Eindhoven, The Netherlands) operating at 120kV. Micrographs were acquired with a Ceta 16M CCD camera (FEI, Eindhoven, The Netherlands) using TEM Image and Analysis software ver. 4.17 (FEI, Eindhoven, The Netherlands). At least 10 spores were imaged for each experimental condition.

## Supplementary Information

**Additional file 1.**

## Data Availability

The datasets used and/or analysed during the current study are available from the corresponding author on reasonable request.

## Abbreviations

BHIS: Brain heart infusion supplemented
CaDPA: Calcium dipicolinic acid
CRA: Chlorine-releasing agent
HCAI: Healthcare-acquired infection
LTRS: Laser tweezers Raman spectroscopy
PCA: Principal component analysis
