# Supplementary material for: Hypervirulent R20291 Clostridioides difficile spores show disinfection resilience to sodium hypochlorite despite structural changes
Source: BMC Microbiol. 2023 Mar 6;23:59. doi: 10.1186/s12866-023-02787-z (PMC9986864; doi:10.1186/s12866-023-02787-z)
Supplement: Supplementary file 1 — Additional file 1. [file 12866_2023_2787_MOESM1_ESM.pdf]

Hypervirulent R20291 *Clostridioides difficile* spores show disinfection resilience to sodium hypochlorite despite structural changes

Supporting information

Dmitry Malyshev[†,*], Imogen Anne Jones[‡], Matthew McKracken[‡], Rasmus Öberg[†], Glenn Harper[‡], Lovleen Tina Joshi[‡] and Magnus Andersson[†,§,*]

[†]Department of Physics, Umeå University, Umeå, Sweden

[‡]Faculty of Health, University of Plymouth, Plymouth

[§]Umeå Centre for Microbial Research (UCMR), Umeå University, Umeå, Sweden

[*]Corresponding authors Dmitry Malyshev and Magnus Andersson
Email: dmitry.malyshev@umu.se and magnus.andersson@umu.se

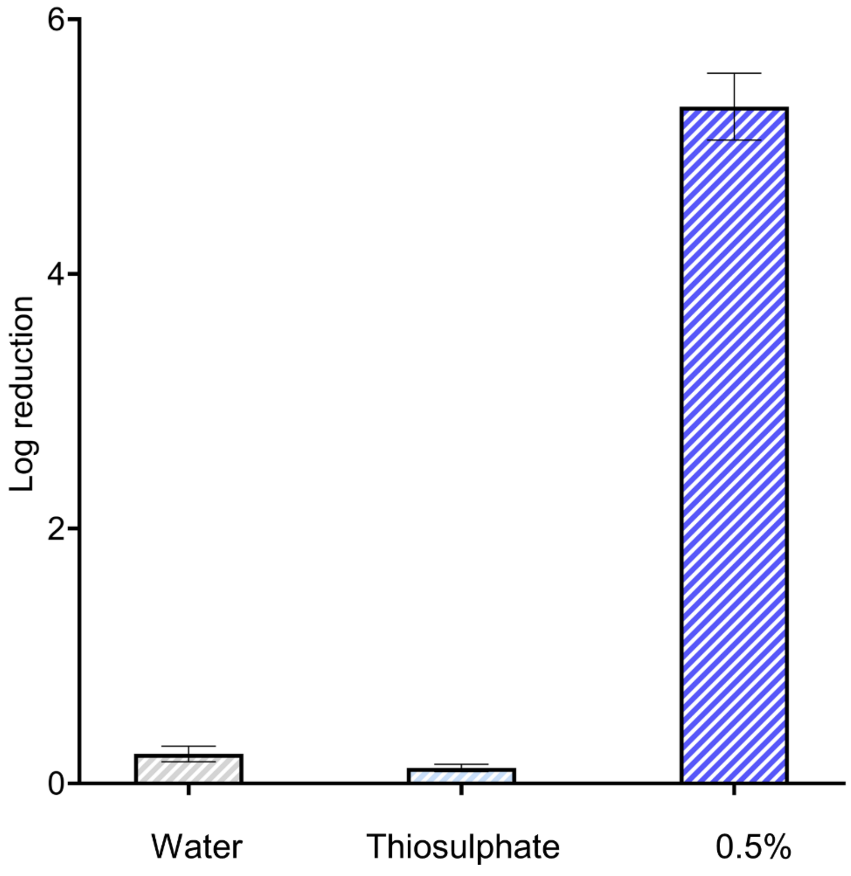

Figure S1. Viability of C. difficile DS1813 spores following hypochlorite treatment. Controls with water and thiosulphate alone are also shown.

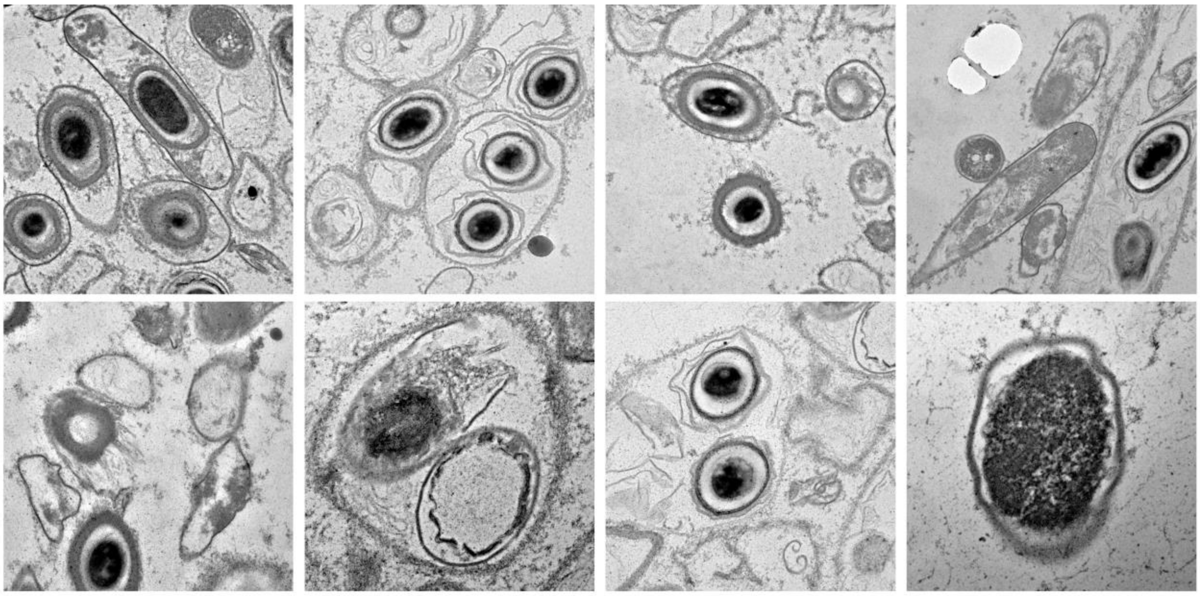

Figure S2. Additional representative TEM images of *C. difficile* R29291 spores treated with 1.0 % sodium hypochlorite for 10 min.

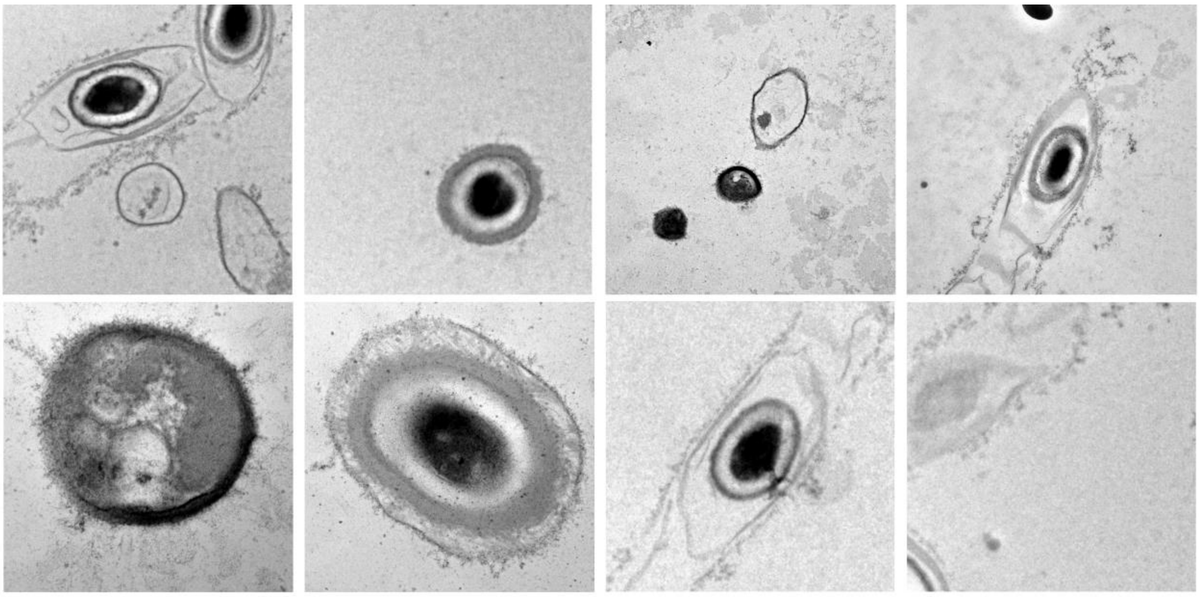

Figure S3. Additional representative TEM images of *C. difficile* CD630 spores treated with 1.0 % sodium hypochlorite for 10 min.

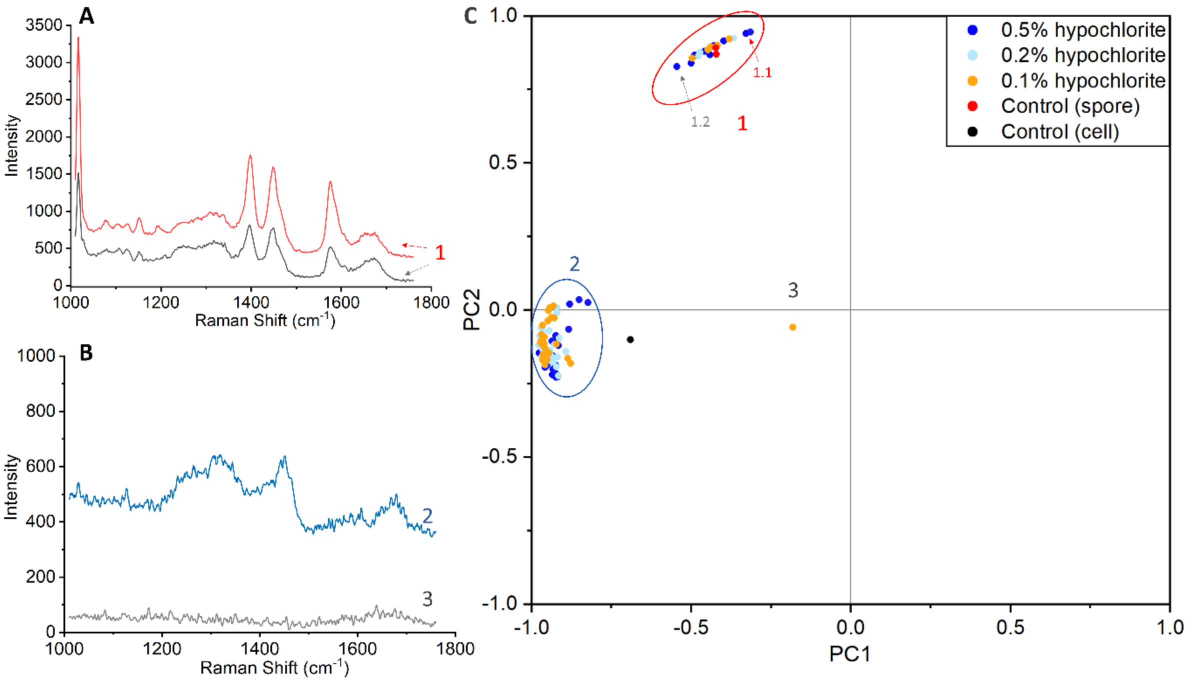

Figure S4. Principal component analysis (PCA) of sodium hypochlorite treated spores in the 1000 - 1700 cm^{-1} spectral range (n=92, including 2 averaged control spectra). The representative observed spectra of spores with CaDPA (A) and without CaDPA (B) are shown. The labelling of the spectra corresponds to the groups in the loadings plot (C).

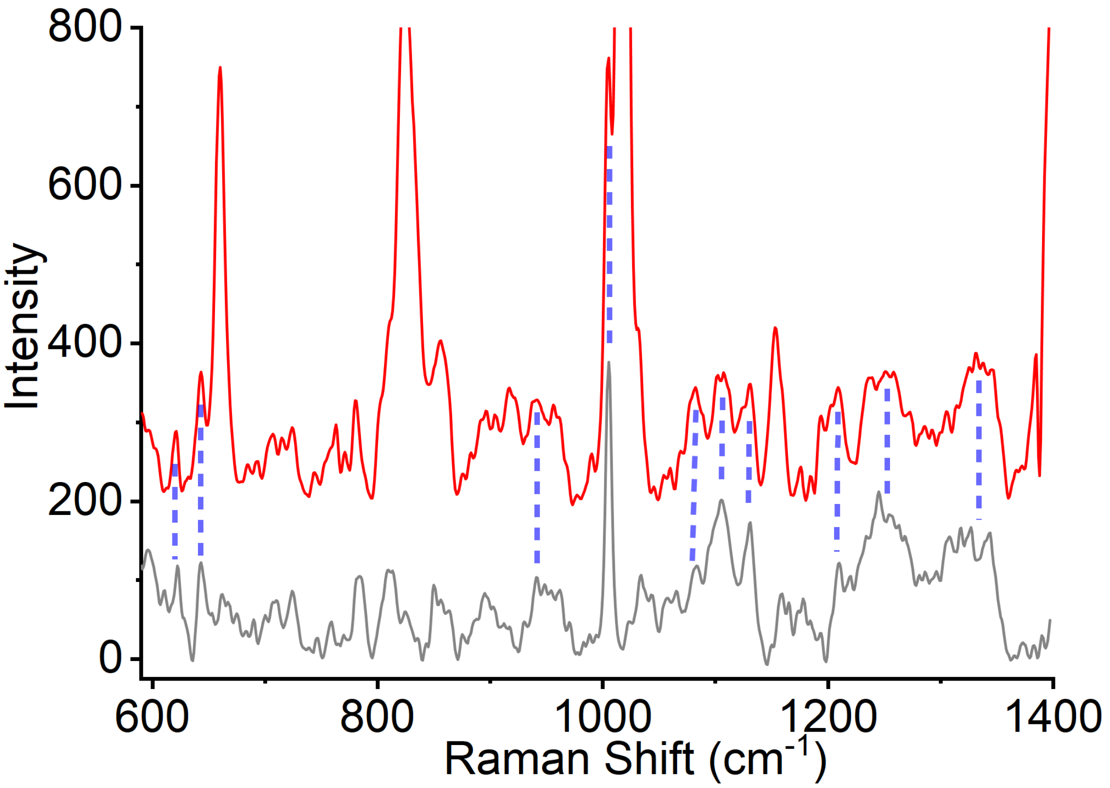

Figure S5. Rescaled plots from Figure 2A-B, highlighting the similarities in the spectra between Group 1 and Group 3 (dashed blue lines), with the exception of the large CaDPA associate peaks.

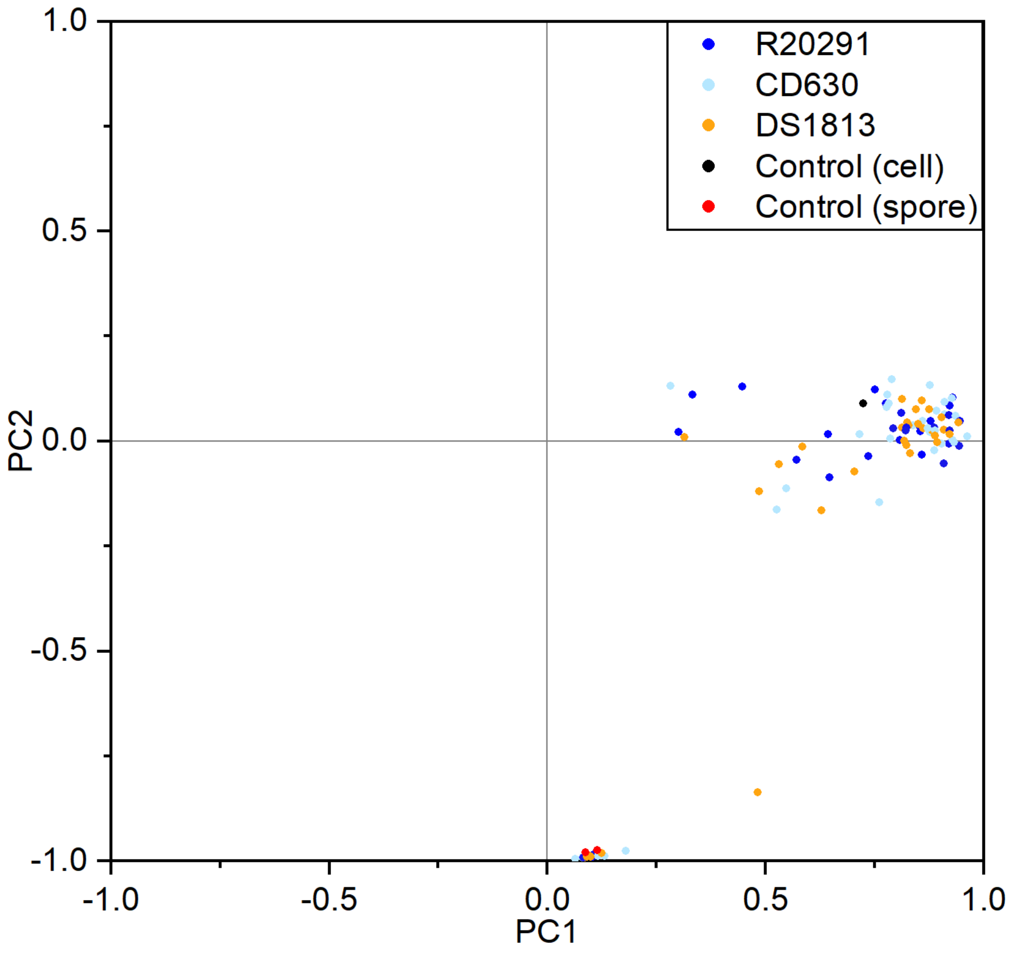

Figure S6. Principal component analysis of sodium hypochlorite treated spores (Figure 2C in main manuscript). Data points recolored based on clinical isolate.

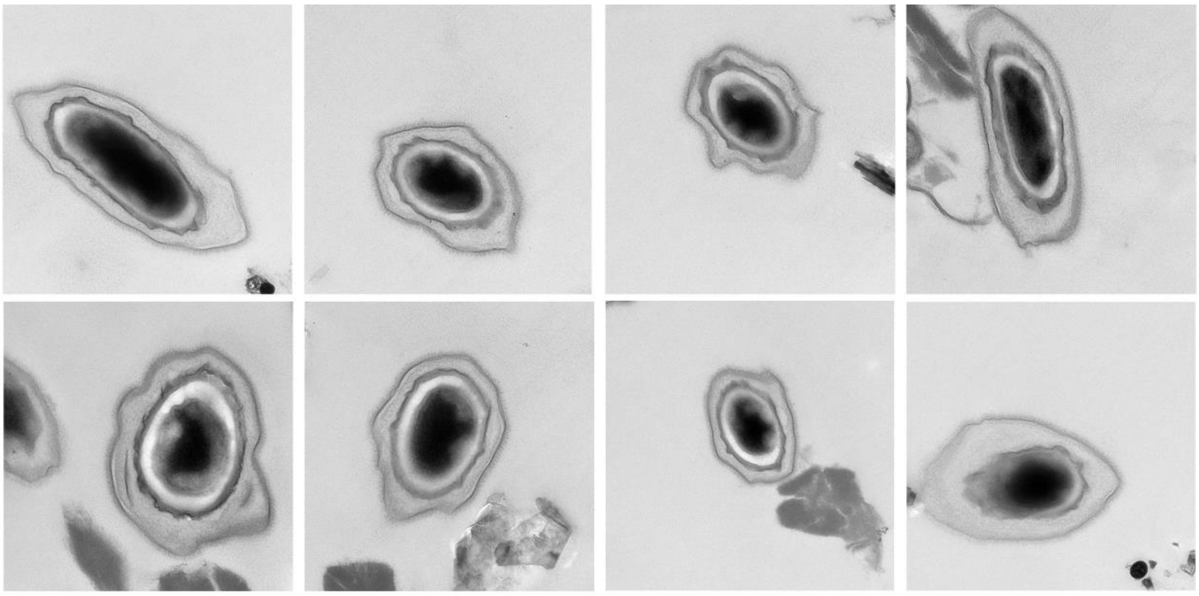

Figure S7. Additional representative TEM images of an untreated *B. thuringiensis* spores.

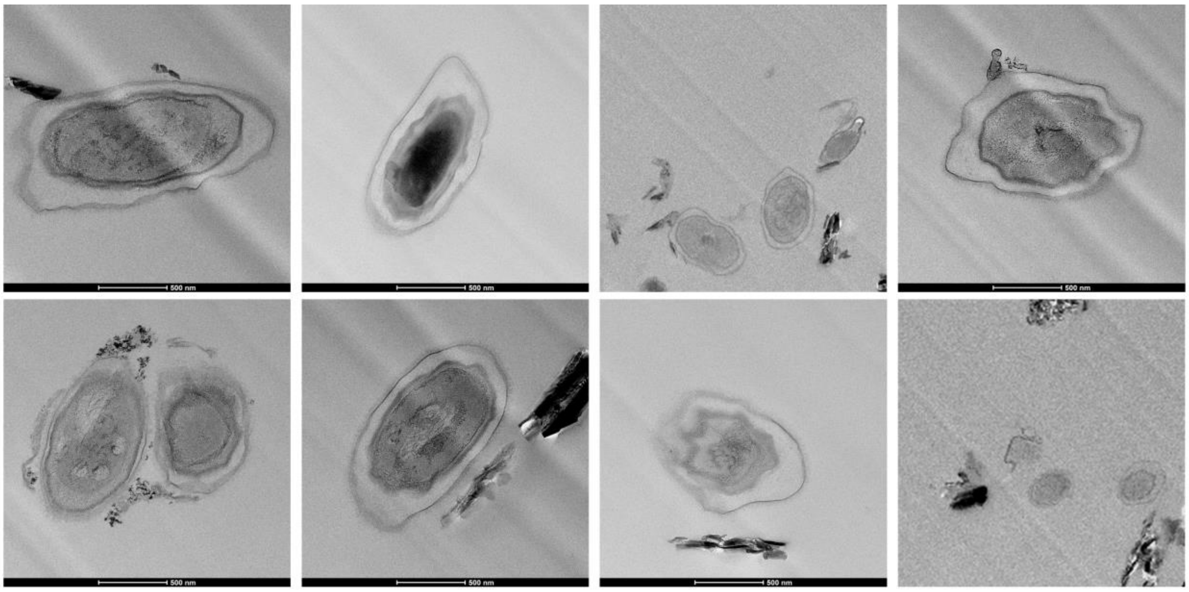

Figure S8. Additional representative TEM images of *B. thuringiensis* spores treated with 0.5% sodium hypochlorite for 10 min.

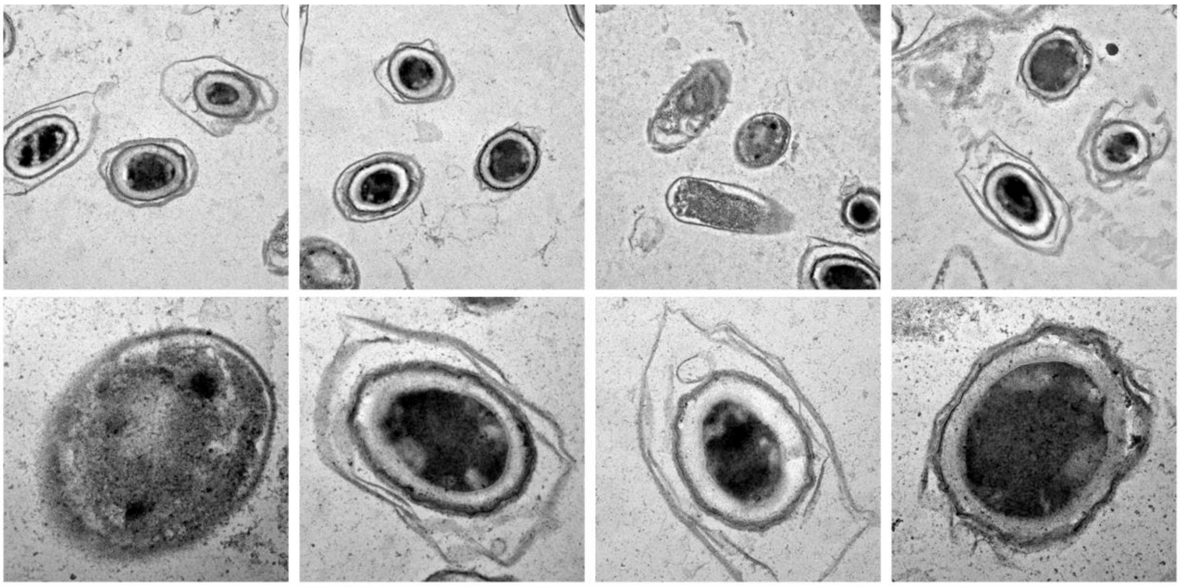

Figure S9. Additional representative TEM images of *C. difficile* R29291 spores treated with 0.5% sodium hypochlorite for 10 min.

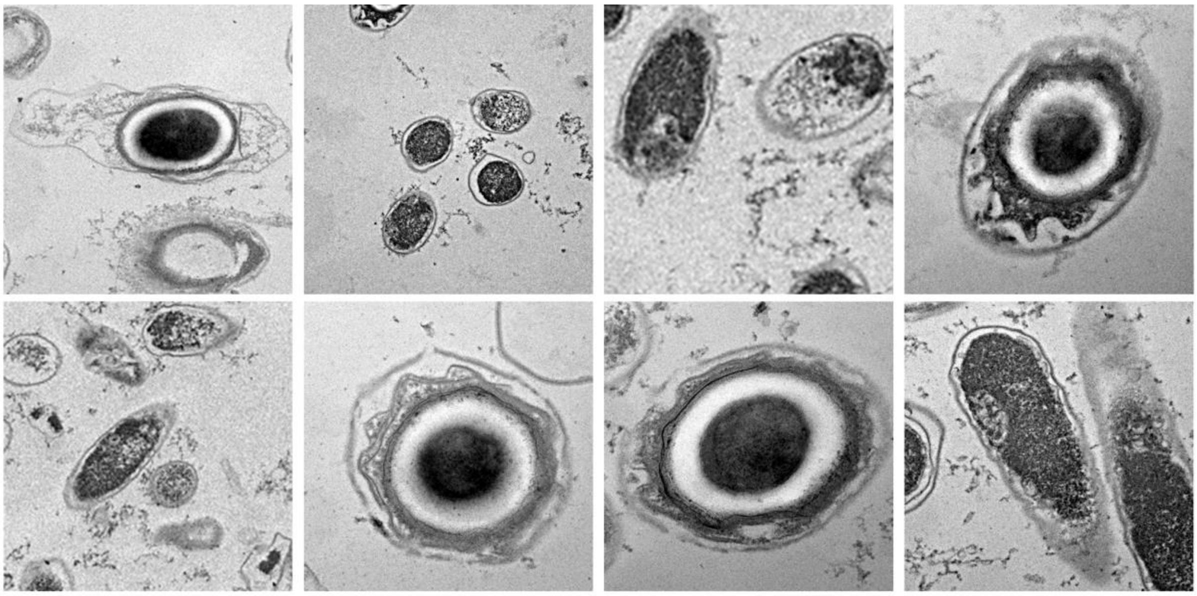

Figure S10. Additional representative TEM images of *C. difficile* CD630 spores treated with 0.5% sodium hypochlorite for 10 min.
